# Complement Activation in Kidneys of Patients With COVID-19

**DOI:** 10.3389/fimmu.2020.594849

**Published:** 2021-01-29

**Authors:** Frederick Pfister, Eva Vonbrunn, Tajana Ries, Hans-Martin Jäck, Klaus Überla, Günter Lochnit, Ahmed Sheriff, Martin Herrmann, Maike Büttner-Herold, Kerstin Amann, Christoph Daniel

**Affiliations:** ^1^ Department of Nephropathology, Institute of Pathology, Friedrich-Alexander-University (FAU) Erlangen-Nürnberg, Erlangen, Germany; ^2^ Nikolaus-Fiebinger-Center FAU, Department of Medicine 3, Division of Molecular Immunology, Friedrich-Alexander-University (FAU) Erlangen-Nürnberg, Erlangen, Germany; ^3^ Department of Virology, Friedrich-Alexander-University (FAU) Erlangen-Nürnberg, Erlangen, Germany; ^4^ Department of Biochemistry, Division Protein Analystics, Justus-Liebig-University Giessen, Giessen, Germany; ^5^ Pentracor GmbH, Henningsdorf, Germany; ^6^ Department of Medicine 3, Institute for Rheumatology and Immunology, Friedrich-Alexander-University (FAU) Erlangen-Nürnberg, Erlangen, Germany

**Keywords:** complement—immunological term, COVID-19, kidney, endothelial injury, lectin pathway activation

## Abstract

Most patients who became critically ill following infection with COVID-19 develop severe acute respiratory syndrome (SARS) attributed to a maladaptive or inadequate immune response. The complement system is an important component of the innate immune system that is involved in the opsonization of viruses but also in triggering further immune cell responses. Complement activation was seen in plasma adsorber material that clogged during the treatment of critically ill patients with COVID-19. Apart from the lung, the kidney is the second most common organ affected by COVID-19. Using immunohistochemistry for complement factors C1q, MASP-2, C3c, C3d, C4d, and C5b-9 we investigated the involvement of the complement system in six kidney biopsies with acute kidney failure in different clinical settings and three kidneys from autopsy material of patients with COVID-19. Renal tissue was analyzed for signs of renal injury by detection of thrombus formation using CD61, endothelial cell rarefaction using the marker E-26 transformation specific-related gene (ERG-) and proliferation using proliferating cell nuclear antigen (PCNA)-staining. SARS-CoV-2 was detected by *in situ* hybridization and immunohistochemistry. Biopsies from patients with hemolytic uremic syndrome (HUS, n = 5), severe acute tubular injury (ATI, n = 7), zero biopsies with disseminated intravascular coagulation (DIC, n = 7) and 1 year protocol biopsies from renal transplants (Ctrl, n = 7) served as controls. In the material clogging plasma adsorbers used for extracorporeal therapy of patients with COVID-19 C3 was the dominant protein but collectin 11 and MASP-2 were also identified. SARS-CoV-2 was sporadically present in varying numbers in some biopsies from patients with COVID-19. The highest frequency of CD61-positive platelets was found in peritubular capillaries and arteries of COVID-19 infected renal specimens as compared to all controls. Apart from COVID-19 specimens, MASP-2 was detected in glomeruli with DIC and ATI. In contrast, the classical pathway (i.e. C1q) was hardly seen in COVID-19 biopsies. Both C3 cleavage products C3c and C3d were strongly detected in renal arteries but also occurs in glomerular capillaries of COVID-19 biopsies, while tubular C3d was stronger than C3c in biopsies from COVID-19 patients. The membrane attack complex C5b-9, demonstrating terminal pathway activation, was predominantly deposited in COVID-19 biopsies in peritubular capillaries, renal arterioles, and tubular basement membrane with similar or even higher frequency compared to controls. In conclusion, various complement pathways were activated in COVID-19 kidneys, the lectin pathway mainly in peritubular capillaries and in part the classical pathway in renal arteries whereas the alternative pathway seem to be crucial for tubular complement activation. Therefore, activation of the complement system might be involved in the worsening of renal injury. Complement inhibition might thus be a promising treatment option to prevent deregulated activation and subsequent collateral tissue injury.

## Introduction

Patients who became critically ill due to SARS-Cov2 virus infection developed severe acute respiratory distress syndrome (ARDS). ARDS is induced by damage of lung alveoli by endothelial injury followed by a maladaptive immune response (1). Tissue damage is not restricted to the respiratory tract since patients with severe COVID-19 often developed multiorgan failure including cardiac and acute kidney injury (2, 3). In a consecutive study of 701 patients with COVID-19 hospitalized in Wuhan, 44% and 27% had proteinuria and hematuria on admission, respectively (4). Furthermore, kidney disease in COVID-19 patients was associated with in-hospital mortality (3). While some reports suggested a direct viral infection of the kidney with infection of glomerular (5) and tubular cells (6–8) other studies support indirect pathomechanisms (9–11). Histopathological findings in postmortem renal biopsies (8, 10, 11) or native and allograft kidney biopsies (9) reported a wide spectrum of glomerular and tubular injury. However, by far the most common renal complication is acute kidney injury (AKI) (8–11). Additionally, scarce focal kidney fibrin thrombi occur (11). The pathophysiology of COVID-19–induced kidney damage is not well understood, but is primarily a result of the host immune response driving hypercytokinemia and aggressive inflammation (12, 13). The cytokine storm that causes lung injury associated with infections is reportedly driven by factors of the complement system (14), which as part of the innate immune system plays an important role in defense against infections. Complement opsonizes viruses or bacteria, activates and attracts leukocytes and lyses bacteria and cells (15). The complement system can be activated by three different pathways, (i) the classical pathway, activated by any structure that is recognized by C1q (16), (ii) the lectin pathway, activated when mannan-binding lectin-associated serine protease 2 (MASP-2) complexes with mannose-binding lectin (MBL), ficolins, or collectin-11 bound to saccharide patterns expressed on bacteria or cells (17, 18) and (iii) the alternative pathway, activated through spontaneous hydrolysis of C3 (19). All complement activation pathways form C3 convertases, which finally initiate the formation of the C5 convertase, which leads to the assembly of C5b-9, the terminal membrane attack complex. In addition, cleavage products C3a and C5a serve as anaphylatoxins that activate and attract leukocytes (15). Since complement activation could be detected in plasma (20), lung and skin (21) from COVID-19 patients and seems to be involved in promoting inflammatory processes that lead to tissue damage in COVID-19 patients we here investigated complement activation in six renal biopsies and three postmortem kidneys from patients with COVID-19. Complement involvement was compared to renal biopsies of acute tubular injury (ATI), known kidney diseases with distinct endothelial cell injury including hemolytic uremic syndrome (HUS) and disseminated intravascular coagulation (DIC) and a control group of 1 year protocol biopsies from stable renal transplants (Ctrl).

## Materials and Methods

### Analysis of Plasma Adsorber–Bound Proteins

Plasma adsorbers (Pentracor CRP, Pentracor, Henningsdorf, Germany) that clogged during the treatment of critically ill COVID-19 patients were analyzed to study proteins involved in this plasma reaction. A sample of 0.5 ml agarose (plasma adsorber material) was washed with 25 ml of 0.9% NaCl solution to remove unbound proteins. After that, 0.5 ml of 2 mg/ml DNase I (Roche, in 10 mM Tris HCl, 2.5 mM MgCl_2_ and 0.5 mM CaCl_2_, pH 7.6) was added and the mixture was incubated for 30 min at 37°C on a tilt shaker. The DNase I and released proteins were eluted with 5 ml PBS, boiled for 15 min in reducing SDS-buffer (10% SDS in 250 mM Tris pH 6.8 supplemented with 2.83 M mercaptoethanol) and then separated by gradient SDS polyacrylamide gel electrophoresis (4–20% Acrylamid). Bands of interest were excised from gels and the proteins were digested with trypsin (10 ng/µl sequencing grade Promega, Mannheim). Tryptic peptides were eluted from the gel slices with 1% trifluoric acid.

### Matrix-Assisted Laser-Desorption Ionization Time-of-Flight Mass Spectrometry (MALDI-TOF-MS) and Protein Identification

MALDI-TOF-MS was performed on an Ultraflex TOF/TOF mass spectrometer (Bruker Daltonics, Bremen) equipped with a nitrogen laser and a LIFT-MS/MS facility. The instrument was operated in the positive-ion reflectron mode using 2.5-dihydroxybenzoic acid and methylendiphosphonic acid as matrix. Sum spectra consisting of 200–400 single spectra were acquired. For data processing and instrument control the Compass 1.4 software package consisting of FlexControl 4.4, FlexAnalysis 3.4 4, Sequence Editor and BioTools 3.2 and ProteinScape 3.1. were used. External calibration was performed with a peptide standard (Bruker Daltonics).

Proteins were identified by MASCOT peptide mass fingerprint search (http://www.matrixscience.com) using the Uniprot Human database (version 20200226, 210438 sequence entries; p<0.05). For the search, a mass tolerance of 75 ppm was allowed and oxidation of methionine as the variable modification was used.

### Human Renal Tissue Specimens

To evaluate the relevance of complement in mediation of renal pathological changes during COVID-19 we used six formalin-fixed paraffin-embedded (FFPE) kidney biopsies of patients with COVID-19 and renal insufficiency in different clinical settings (four transplant kidneys, one ANCA-associated vasculitis, one multiple organ dysfunction syndrome) and three FFPE autopsy kidneys of patients who died with multiple organ dysfunction syndrome following SARS-CoV-2 infection. Kidney biopsies of patients with hemolytic uremic syndrome (HUS, n = 5), severe acute tubular injury due to septic shock (ATI n = 7), zero-biopsies with disseminated intravascular coagulation (DIC, n = 7) and 1 year protocol biopsies from stable renal transplants (Ctl; n = 7) served as controls. All tissue samples were collected from the archive of the Department of Nephropathology, Friedrich-Alexander-University Erlangen-Nuremberg and analysis was approved by the local Ethics committee (reference number 4415). Patient characteristics of the investigated cases with COVID-19 are described in **Table 1**.

**Table 1:** Clinical, laboratory, and histopathological findings in patients with COVID-19 infection.

Pt	Age	Sex	Biopsy indication	Comorbidities/circumstances	SARS-CoV-2 infection	PU	Serumcreatinine[mg/dl]	Bx diagnosis
**1**	40	M	AKI, PU, HU, ANCA positivity	HTN	Current	1 g/g	3,1	ANCA-asso. Crescentic GN, ATN
**2**	76	M	MODS, AKI	DM	Current	++	7,5	ATN
**3**	69	M	AKI, ESKD, RTx 2007	DM, HTN, acute enteritis	Current	0,7 g/g	2,1	ATN
**4**	52	F	AKI, progress PU, ESKD, RTx 2011	DM, HTN	Current	1,5 g/g	1,5	ATN
**5**	70	M	AKI, ESKD, RTx	Bacterial pneumonia	Current	++	1,4	ATN, infect-asso.GN, cellular borderline changes
**6**	35	M	AKI, progress PU, ESKD, RTx 2019	–	Current	1,5 g/g	2,1	ATN, recurrent FSGS
**7**	41	F	ARDS, MODS, AKI, post-mortem	Pregnancy (32gw)	Current	Nd	Nd	ATN
**8**	64	M	ARDS, MODS, AKI, post-mortem	HTN	Current	Nd	Nd	ATN
**9**	71	M	ARDS, MODS, AKI, LE, post-mortem	HTN	Current	Nd	Nd	ATN

Pt, patient; M, male; F, female; PU, proteinuria measured as g proteinuria/g creatinine or dipstick; HU, hematuria; AKI, acute kidney insufficiency; ESKD, end-stage kidney disease; RTx, renal transplantation; ARDS, acute respiratory distress syndrome; MODS, multiple organ dysfunction syndrome; HTN, hypertension; DM, diabetes mellitus; ATN, acute tubular necrosis; FSGS, focal-segmental glomerulosclerosis; ANCA, anti-neutrophilic cytoplasmatic antibody; GN, glomerulonephritis; Nd, unknown.

### In Situ Hybridization

In situ hybridization, for detection of SARS-CoV-2 RNA in FFPE tissue, was performed using a V nCoV2019-S probe (848561; ACD, Hayward, CA, USA), specific for the S gene encoding the spike protein from SARS-CoV-2, and the RNAscope 2.5 HD RED Kit (ACD). Tissue sections of 4 µm thickness were deparaffinized in xylene, dehydrated in ethanol and blocked with peroxidase. Slides were boiled in kit-provided antigen retrieval buffer at 95°C for 15 min and digested afterwards with protease at 40°C for 30 min. The target probe were hybridized with kidney sections in the HybEZ hybridization oven (ACD) at 40°C for 2 h. Pre-amplification and amplification steps were conducted using kit-provided reagents according to the manufacturer’s recommendations. For signal detection sections were incubated with Fast Red substrate for 10 min at room temperature followed by counterstaining with hematoxylin, drying at 60°C for 15 min and mounting with Eco Mount (ACD). The same procedure was used for *in situ* hybridization with the Hs-COLEC11 probe (542431; ACD) for the detection of human collectin 11 mRNA.

### Immunohistochemistry

For immunohistochemical stainings formalin-fixed paraffin-embedded (FFPE) kidney biopsies were cut into 2 µm sections, deparaffinized and rehydrated. Antigen retrieval was performed using pronase E (Sigma Aldrich, Taufkirchen, Germany) digestion for 30 min at 37°C (C1q, C3c, C5b-9) or cooking in target retrieval solution pH 6 (DAKO Deutschland GmbH, Hamburg, Germany) for 2.5 min (C3d, C4d, MASP-2, CD61, COVID-19 spike protein). Endogenous peroxidase was blocked with 3% H_2_O_2_ and unspecific antigens with Avidin-Biotin (Vector laboratories, Burlingame, CA, USA) and normal goat or horse serum in blotto (1:5). The following primary antibodies were diluted in 50 mM Tris pH 7,4 and incubated over-night at 4°C or for 1 h at room temperature: C1q, a rabbit polyclonal antibody against human C1q (A0136; DAKO Deutschland GmbH); C3c, a rabbit polyclonal antibody against human C3c (A0062; DAKO Deutschland GmbH); C3d, a rabbit monoclonal antibody against human C3d (ab136916; Abcam, Cambridge, UK); C5b-9, a mouse monoclonal antibody against human C5b-9 (M0777; DAKO Deutschland GmbH); MASP-2, a rabbit polyclonal antibody against human Mannan-binding lectin serine peptidase 2 (HPA029313; Sigma Aldrich); CD61 a mouse monoclonal antibody against human platelet glycoprotein IIIa (M0753; DAKO Deutschland GmbH), C4d, a rabbit polyclonal antibody against human C4d (BI-RC4D; Biomedica, Vienna, Austria) and SARS-CoV-2 spike protein, a mouse monoclonal antibody against SARS-CoV-2 spike protein (clone 224.2, kindly provided by M.-H. Jäck, Department of Molecular Immunology, FAU Erlangen-Nürnberg). After washing with 50 mM Tris pH 7.4, sections were incubated with biotinylated secondary goat anti-rabbit IgG (BA-1000; Vector laboratories) or horse anti-mouse IgG (BA-2001, Vector laboratories). Detection of bound antibodies was conducted using ABC-Kit and DAB-Impact as a substrate (both from Vector laboratories), while nuclei were counter stained with hematoxylin. For negative controls primary antibody was substituted by antibody dilution buffer (50 mM Tris pH 7,4) (**Supplemental Figure 3**). An overview with representative stainings of all cases is shown in **Supplemental Figure 2**.

### Immunofluorescence Double Staining

For evaluation of endothelial cell injury and proliferative activity we performed immunofluorescence double staining on rehydrated FFPE kidney sections using a rabbit anti-human E26-transformation- specific related gene (ERG) antibody for detection of endothelial cells (diluted 1:100 in 50 mM Tris pH 7,4; EP111, Cell Marque, Rocklin, CA, USA) and a mouse anti-human proliferating cell nuclear antigen (PCNA, M0879, diluted 1:1000 in 50 mM Tris pH 7.4; DAKO Deutschland GmbH, Hamburg Germany). After dewaxing, sections were incubated with primary antibodies over-night at 4°C followed by washing with 50 mM Tris pH 7.4 supplemented with 0.05%Tween 20. A donkey anti-rabbit IgG Alexa Fluor 568 and a donkey anti-mouse IgG Alexa633 (both from Thermo Fisher Scientific, Waltham, MA, USA) were used as secondary antibodies diluted 1:200 in 50 mM Tris pH 7.4 and incubated at room temperature for 30 min. After subsequent washing, cell nuclei were stained with DAPI (diluted 0.2 µg/ml in distilled water) for 5 min, followed by rinsing in Tris buffer. Finally, sections were covered with Mowiol mounting medium (Calbiochem, La Jolla, USA) and analyzed using laser scanning confocal microscopy (LSM Zeiss 710) and quantification of fluorescence positive area by Zen software (Zeiss GmbH, Jena, Germany).

### Semi-Quantitative Evaluation of Complement and CD61 in Renal Biopsies

Complement staining in renal biopsies was graded in different vascular compartments of the kidney (i.e. in the glomeruli, peritubular capillaries and arteries), using a semi-quantitative immunohistochemical staining score (score 0, 1 or 2), which describes the distribution and intensity of staining signal in the micro- and macrovascular structures. In detail for CD61, score 0 was defined as no positive thrombocytes within vascular lumina, score 1 was defined as single/scattered positive thrombocytes in vascular lumina and score 2 was defined as presence of intravascular thrombocyte aggregates of any size. For the components of the complement system (C1q, C3c, C3d, C4dC5b-9, MASP-2) score 0 was defined as no specific granular reactivity along endothelial cell surfaces in the different vascular compartments or tubular basement membrane, score 1 was defined as minimal reactivity in single glomeruli and peritubular capillaries and mild reactivity along endothelial cells or in the intima of arteries and focal reactivity along the tubular basement membrane and score 2 was defined as reactivity in > 10% to 20% of glomeruli and peritubular capillaries and moderate reactivity in the majority (>50%) of arteries and diffuse clear reactivity along tubular basement membranes.

### Transient Expression of SARS-CoV-2 Spike Protein

Cells producing membrane-anchored CoV-2 spike protein cells were established by co-transfecting HEK 293T with the PEI method with a GFP reporter plasmid and a pCG1-based expression vector for the spike protein of SARS-CoV-2 (position 21580 – 25400 from accession no. NC_045512).

### Generation of Anti-SARS-CoV-2 Spike Protein Ab

The monoclonal IgG2c antibody against the CoV-2 spike protein was isolated from a hybridoma line that was established by the conventional hybridoma technology from spleen cells of Trianni mice that were immunized with DNA-encoding CoV-2 spike protein and purified CoV-2 spike protein. The Trianni mouse line (Patent US 2013/0219535 A1) carried the complete repertoire of human variable region gene segments of immunoglobulin (Ig) heavy (HC) and L chains (LC).

### Statistical Analyses

After testing for normal distribution of values using Kolmogorov-Smirnov test, data were analyzed using Kruskal-Wallis test with Dunn’s Multiple Comparison test as *post hoc* test for comparison of multiple groups. In all tests p<0.05 was accepted as statistically significant. Statistical analyses were performed using GraphPad Prism 8 for Windows software (version 8.3, GraphPad software Inc., San Diego, CA, USA).

## Results

### Complement C3 Was the Main Component in Clotted Plasma Adsorber From Patients With COVID-19

**Figure 1:**
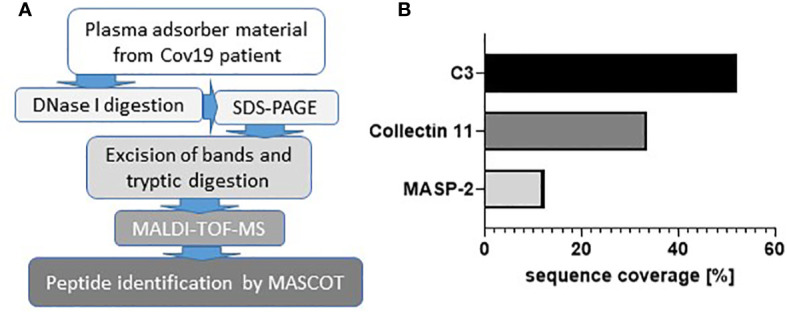
Involvement of complement components in COVID-19. Workflow for the analysis of clotted material isolated from plasma adsorber material collected from treated COVID-19 patient **(A)**. Coverage rates of complement factors C3, collectin 11, and mannose-binding protein-associated serine protease 2 (MASP-2) analyzed by MALDI-TOF **(B)**.

When seriously ill patients with COVID-19 in our clinic were connected to plasma absorption columns for therapeutic purposes, we observed that these columns quickly clogged. From more than 90 patients treated with CRP apheresis with various diseases (including severe myocardial infarction (STEMI), cardiogenic shock, after bypass surgery, severe COVID-19, acute pancreatitis, sepsis, SIRS after failed bypass surgery) the massive clogging of the columns only occurred in patients with severe COVID-19. In patients with heart attack much smaller clots were observed on the sieve filters but not on the adsorber material. To identify the proteins that are involved in this clot-forming reaction of the plasma, the proteins were detached from the adsorber material and sieve filters by treatment with DNase1 and separated by electrophoresis. Individual bands were digested with trypsin and finally examined by MALDI-TOF (**Figure 1A**). First, we analyzed the most prominent bands of an electrophoretic separation in an unbiased manner for the major peptides and detected C3 and C3 fragments with a sequence coverage of 52% (**Figure 1B**). Next, we analyzed the candidate peptides of mannose-binding protein-associated serine protease 2 (MASP-2) and collectin 11 involved in complement activation *via* the lectin pathway and detected both molecules (MASP-2; sequence coverage 12%) and collectin 11 (sequence coverage 33%) (**Figure 1B**). MASP-2. In contrast, the investigation of the clots from treatment of heart attack patients showed haptoglobin as main component and only little C3a (data not shown). This finding might indicate an important role of complement in the pathogenesis of COVID-19 disease and encouraged us to investigate complement activation in the kidneys of COVID-19 patients in more detail.

### Histopathology, Proliferative Activity, and Loss of Endothelial Cells in Kidneys From Patients With COVID-19

**Figure 2:**
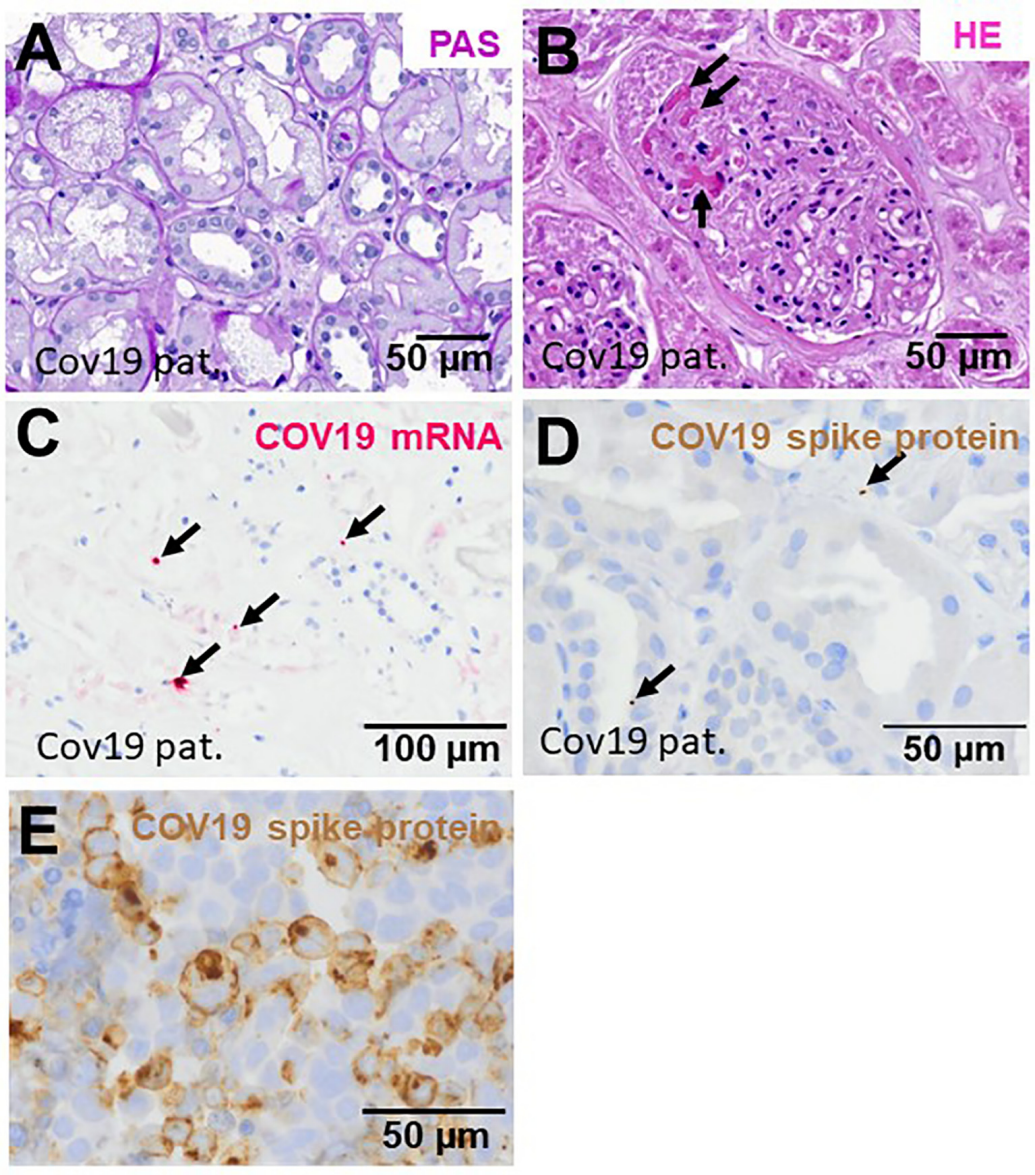
Renal injury in kidney biopsies from patients infected with SARS-CoV-2. Representative pictures of a PAS-stained COVID-19 renal biopsy showing acute tubular injury with swollen and vacuolized cells **(A)** and glomerular microthrombi in HE-stained section (**B**, arrows). In situ hybridization for SARS-CoV-2 mRNA showed few positive signals in tubular and endothelial localization (**C**, pink signals marked by arrows). Immunohistochemistry for SARS-CoV-3 spike protein detected sporadic positive signals in renal biopsies from patients with COVID-19 (**D**, brown signals marked by arrows). As positive control, we employed HEK293T cells transfected with a plasmid expressing SARS-CoV-2 spike protein, fixed with formalin, embedded in paraffin followed by staining of sections by immunohistochemistry using anti-SARS-CoV-2 spike antibody (**E**, brown staining).

**Figure 3:**
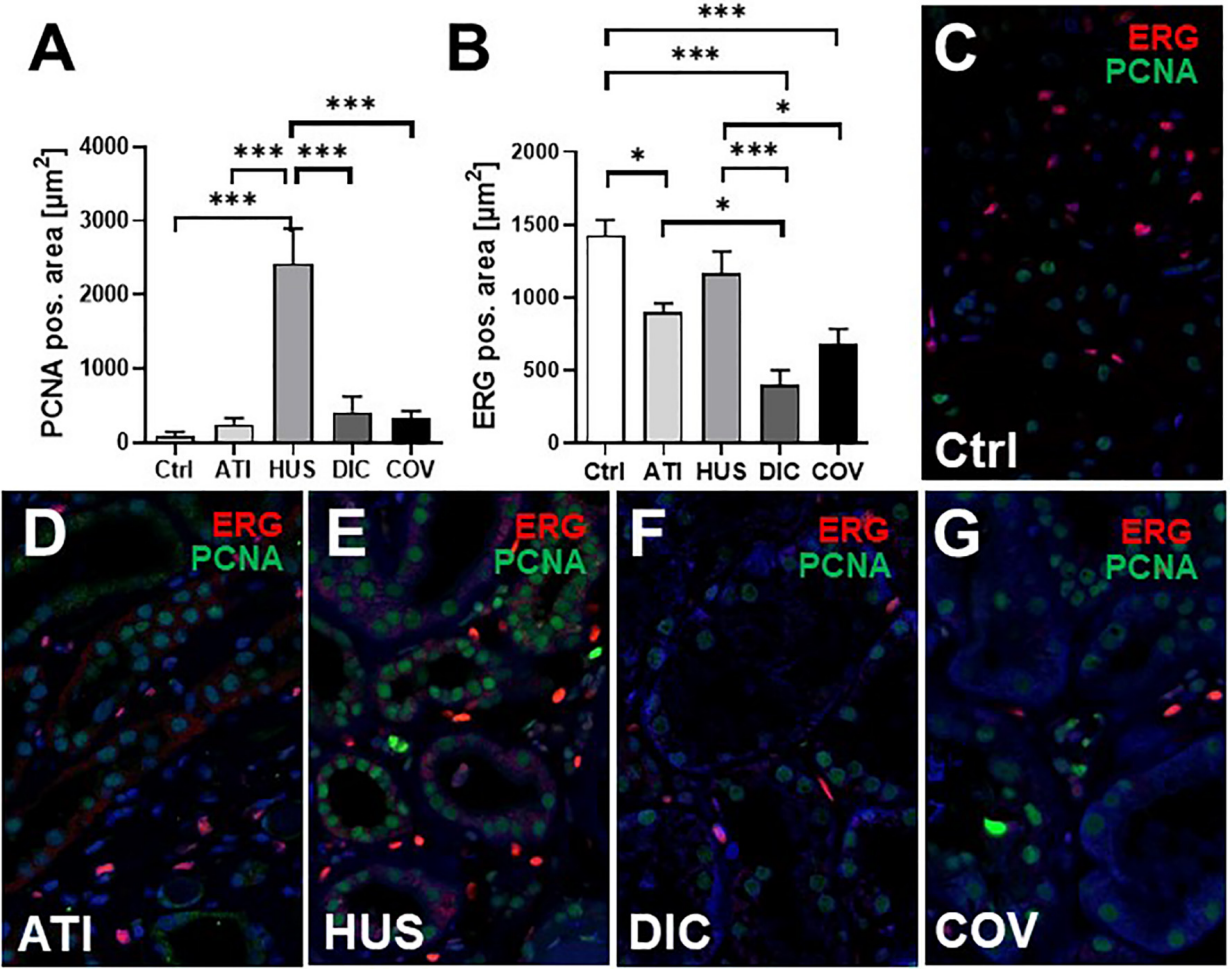
Endothelial rarefaction and proliferative activity in kidney biopsies from patients infected with SARS-CoV-2. Renal proliferation, as assessed by PCNA-staining **(A)** and ERG-positive endothelial cells **(B)** were evaluated by immunofluorescence staining analyzed by confocal microscopy and quantification by ZEN-software in COVID-19 biopsies (COV, n = 7) compared to renal control biopsies taken 1 year after transplantation (Ctrl, n=7), biopsies with acute tubular injury (ATI, n = 7), hemolytic uremic syndrome (HUS, n = 5) and disseminated intravascular coagulation (DIC, n = 7). Representative pictures of immunofluorescence double staining showing PCNA (green staining) and ERG (red staining) double staining were shown in biopsies of Ctrl **(C)**, ATI **(D)**, HUS **(E)**, DIC **(F)**, and COV **(G)**. *p < 0.05; ***p < 0.001.

In this study, six kidney biopsies of patients with COVID-19 infection (four transplant kidneys, 1 ANCA-associated vasculitis, one multiple organ dysfunction syndrome) and three autopsy kidneys of patients who died with multiple organ dysfunction syndrome following COVID-19 infection were analyzed (**Table 1**). In all tissue samples a moderate to severe acute tubular injury (ATI) with tubular dilatation and vacuolization of swollen tubular epithelial cells was found (**Figure 2A**) and in one of the postmortem tissue samples isolated glomerular microthrombi were present (**Figure 2B**). In addition, an infect-associated glomerulonephritis (most likely related to a bacterial pneumonia the patient developed secondary to the COVID-19) was diagnosed in one proteinuric transplant patient (patient 5), accompanied by acute T-cell mediated borderline-rejection. Another proteinuric transplant patient suffered from recurrence of podocytopathy with focal-segmental glomerulosclerosis in the transplanted kidney and in the biopsy of patient 1 an ANCA-associated crescentic glomerulonephritis was diagnosed. SARS-CoV-2 was sporadically detected by *in situ* hybridization in renal tubules and endothelial cells in six of nine kidney specimens of patients with COVID-19 (**Figure 2C**). However, renal infection with COVID-19 could be confirmed in only one case by immunohistochemistry with an antibody directed against the SARS-CoV-2 spike protein (**Figure 2D**), indicating at least low renal virus load. In contrast, this antibody sensitively recognized paraffin embedded HEK293T cells transfected with SARS-CoV-2 spike protein (**Figure 2E**). To further assess renal damage in patients with COVID-19, we examined proliferating cell nuclear antigen (PCNA) as a marker of increased repair and the expression of the endothelial cell marker ERG in COVID-19 kidney biopsies and compared these to biopsies of kidney diseases characterized by tubular or endothelial damage. Compared to biopsies with hemolytic uremic syndrome (HUS) the kidneys of patients with COVID-19 (COV) showed significantly lower proliferative activity measured as PCNA positive area, which was comparably low to those of biopsies with acute tubular injury (ATI), disseminated intravascular coagulation (DIC) and in 1 year protocol biopsies that served as controls (Ctrl) (**Figures 3A, C–G**, green staining). Capillarization of COVID-19 biopsies, measured as ERG-positive area, was less 50% and 40% than in Ctrl and HUS (**Figures 3B, C, E, G**, red staining). Only DIC showed higher endothelial cell loss while endothelial cell loss in ATI was comparable to COV (**Figures 3B, D, F**).

### Thrombus-Forming CD61-Positive Platelets Were Frequently Detected in Kidneys From Patients With COVID-19

**Figure 4:**
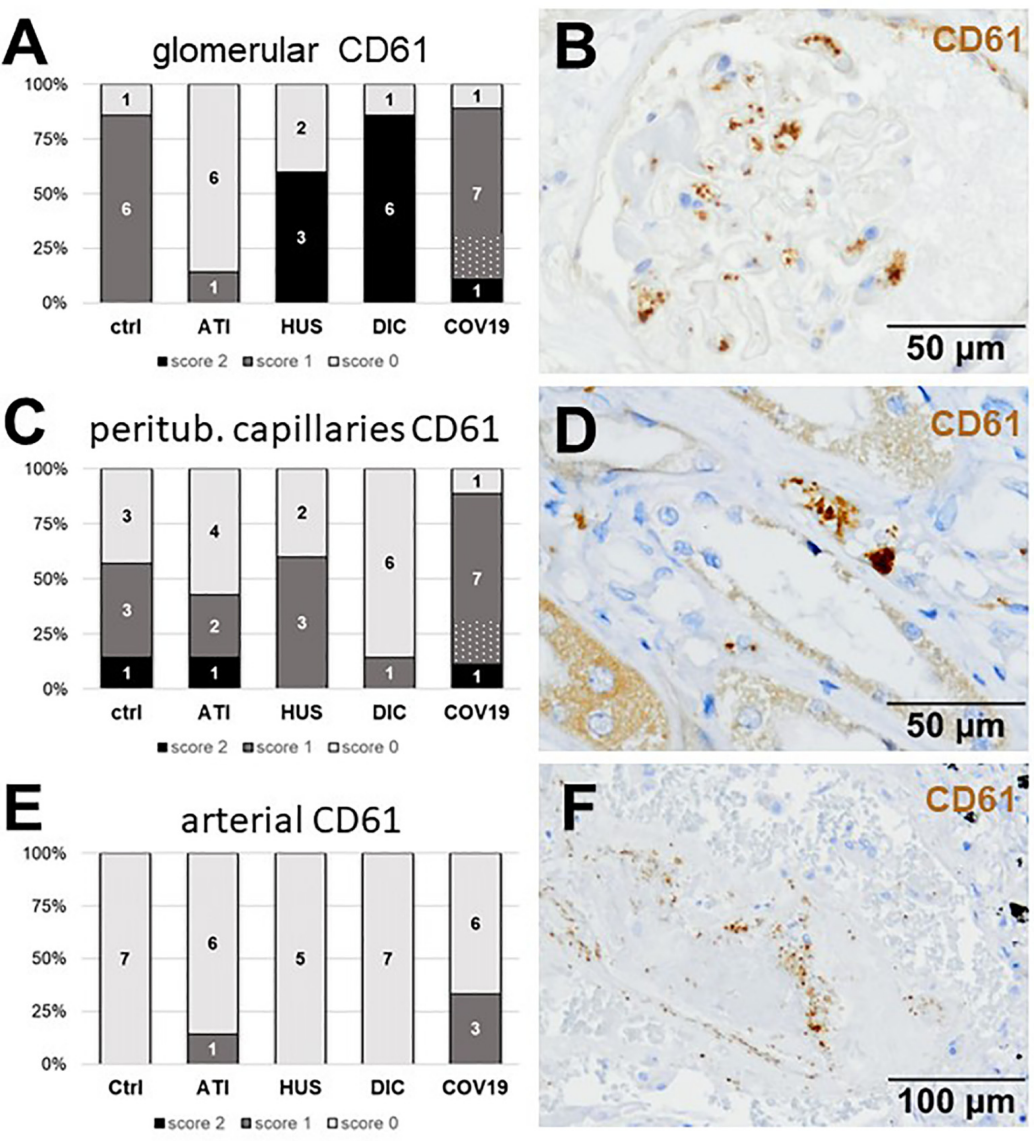
Frequency and localization of CD61-positive platelets in renal biopsies with COVID-19 compared to Ctrl, ATI, HUS, and DIC. Frequency and amount of CD61-positive platelets was analyzed in glomerular capillaries **(A)**, peritubular capillaries **(C)** and renal arteries **(E)** in renal control biopsies taken 1 year after transplantation (Ctrl, n = 7), biopsies with acute tubular injury (ATI, n = 7), hemolytic uremic syndrome (HUS, n = 5), disseminated intravascular coagulation (DIC, n = 7), and COVID-19 (COVID-19, n = 9) using immunohistochemistry and semi-quantitative scoring. The proportion of CD61-positive stained cases that belongs to cases with comorbidities with known involvement of complement activation is marked by hatching in the bars representing the COVID-19 cohort. Representative pictures of COVID-19 biopsies positive for CD61 were shown for glomerular (**B**, brown staining), peritubular (**D**, brown staining), and arterial localization (**F**, brown staining).

First, we investigated CD61-positive platelets in COVID-19 renal biopsies, which are involved in thrombus formation and detected glomerular CD61-positive platelets in 8/9 of the COVID-19 cases (**Figures 4A, B**). Glomerular CD61-positive platelets were comparable in Ctrl and COVID-19, and decreased compared to DIC and HUS glomeruli but ATI had the lowest number of CD61-positive platelets (**Figure 3A**). In peritubular capillaries and renal arteries, CD61-positive platelets counts were higher in COVID-19 biopsies as compared to DIC, HUS and ATI and Ctrl (**Figures 4C–F**). The overall score for CD61, summarizing the scores of all three vascular beds, was highest in COVID-19 compared to all controls (**Table 2**).

**Table 2:** Overview of renal complement deposition in patients with COVID-19 compared to Ctrl, ATI, HUS and DIC.

	Ctrl	ATI	HUS	DIC	COVID-19
**CD61**	0.52	0.29	0.6	0.61	0.78
**MASP-2**	0.07	0.07	0.1	0.2	0.22
**C1q**	0.14	0	0.8	0.79	0.19
**C3c**	0.54	0.89	1	1.25	0.75
**C3d**	0.39	0.48	0.55	0.61	0.89
**C4d**	0	0	0	0	0
**C5b-9**	0.5	0.43	0.65	0.93	1

The mean values of all semi-quantitative compartment-specific scores were summarized and displayed on a heat map.

Ctrl, 1 year renal transplant protocol biopsies; ATI, acute tubular injury; HUS, hemolytic uremic syndrome; DIC, disseminated intravascular coagulation.

### Marked Complement Activation Occurs in Kidney Vascular Beds of Patients With COVID-19

**Figure 5:**
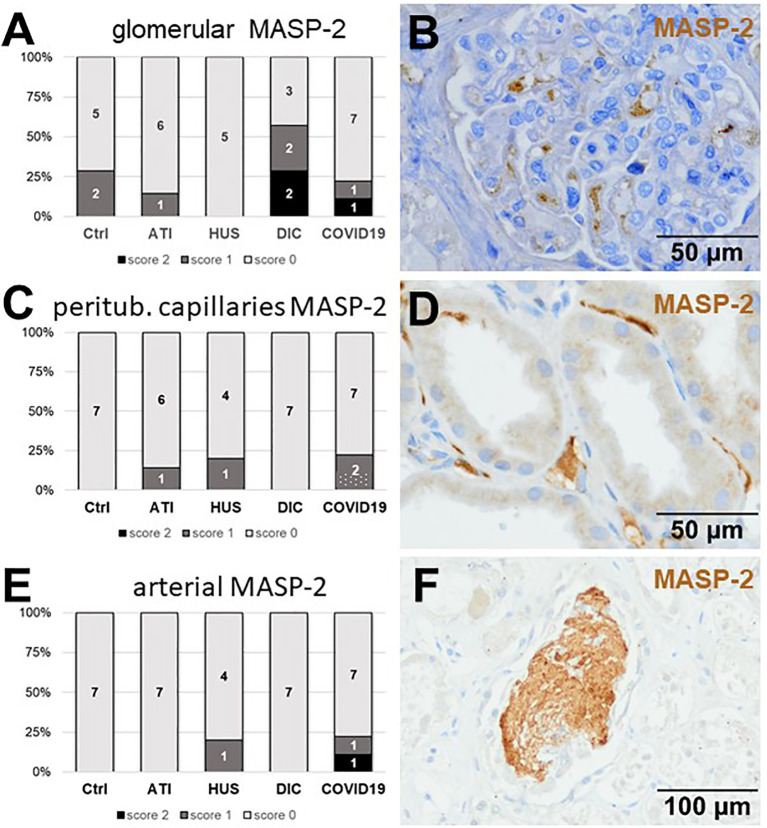
Frequency and vascular localization of MASP-2 deposition in renal biopsies with COVID-19 compared to Ctrl, ATI, HUS, and DIC. Frequency and amount of mannose-binding protein-associated serine protease 2 (MASP-2) deposition was analyzed in glomerular capillaries **(A)**, peritubular capillaries **(C)** and renal arteries **(E)** in renal control biopsies taken 1 year after transplantation (Ctrl, n = 7), biopsies with acute tubular injury (ATI, n = 7), hemolytic uremic syndrome (HUS, n = 5), disseminated intravascular coagulation (DIC, n = 7), and COVID-19 (COVID-19, n = 9) using immunohistochemistry and semi-quantitative scoring. The proportion of MASP-2–positive stained cases that belongs to cases with comorbidities with known involvement of complement activation is marked by hatched bars representing the COVID-19 cohort. Representative pictures of COVID-19 biopsies positive for MASP-2 were shown for glomerular (**B**, brown staining), peritubular (**D**, brown staining) and arterial localization (**F**, brown staining).

**Figure 6:**
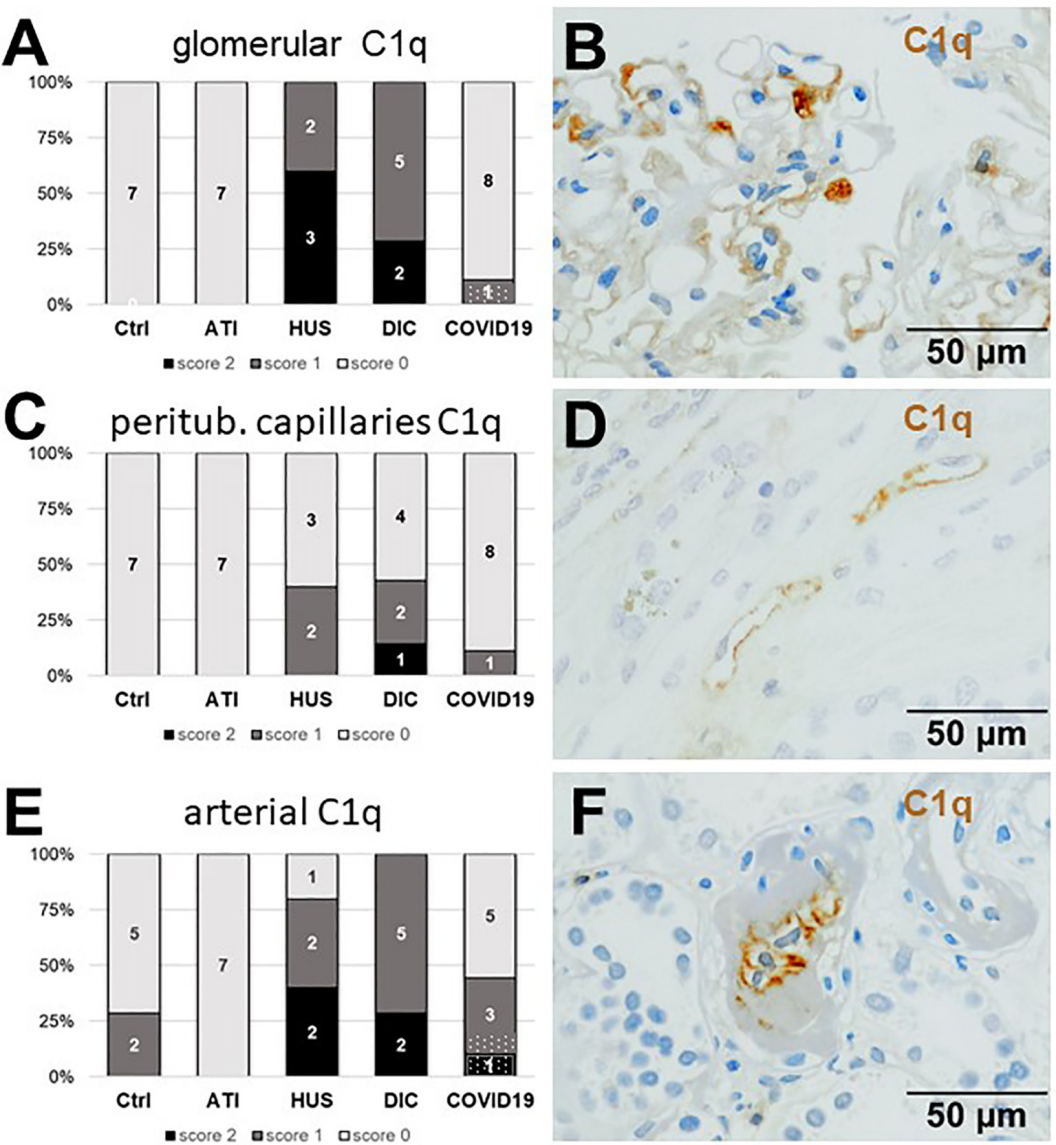
Frequency and vascular localization of C1q deposition in renal biopsies with COVID-19 compared to Ctrl, ATI, HUS, and DIC. Frequency and amount of C1q deposition were analyzed in glomerular capillaries **(A)**, peritubular capillaries **(C)**, and renal arteries **(E)** in renal control biopsies taken 1 year after transplantation (Ctrl, n = 7), biopsies with acute tubular injury (ATI, n = 7), hemolytic uremic syndrome (HUS, n = 5), disseminated intravascular coagulation (DIC, n = 7) and COVID-19 (COVID-19, n = 9) using immunohistochemistry and semi-quantitative scoring. The proportion of C1q-positive stained cases that belongs to cases with comorbidities with known involvement of complement activation is marked by hatched bars representing the COVID-19 cohort. Representative pictures of COVID-19 biopsies positive for C1q were shown for glomerular (**B**, brown staining), peritubular (**D**, brown staining) and arterial localization (**F**, brown staining).

**Figure 7:**
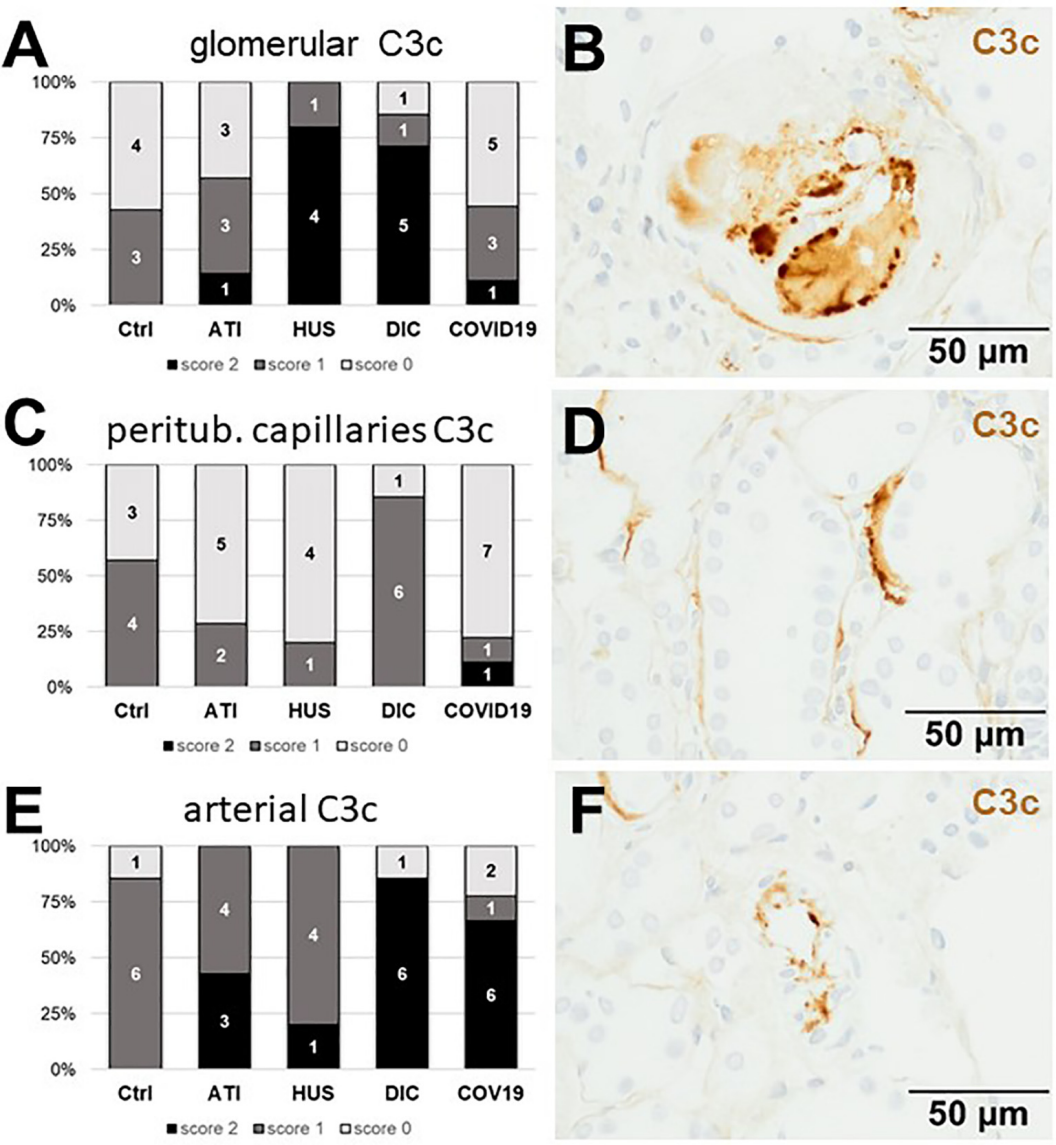
Frequency and vascular localization of C3c deposition in renal biopsies with COVID-19 compared to Ctrl, ATI, HUS and DIC. Frequency and amount of C3c deposition were analyzed in glomerular capillaries **(A)**, peritubular capillaries **(C)**, and renal arteries **(E)** in renal control biopsies taken 1 year after transplantation (Ctrl, n = 7), biopsies with acute tubular injury (ATI, n = 7), hemolytic uremic syndrome (HUS, n = 5), disseminated intravascular coagulation (DIC, n = 7) and COVID-19 (COVID-19, n = 9) using immunohistochemistry and semi-quantitative scoring. The proportion of C3d-positive stained cases that belongs to cases with comorbidities with known involvement of complement activation is marked by hatched bars representing the COVID-19 cohort. Representative pictures of COVID-19 biopsies positive for C3c were shown for glomerular (**B**, brown staining), peritubular (**D**, brown staining) and arterial localization (**F**, brown staining).

**Figure 8:**
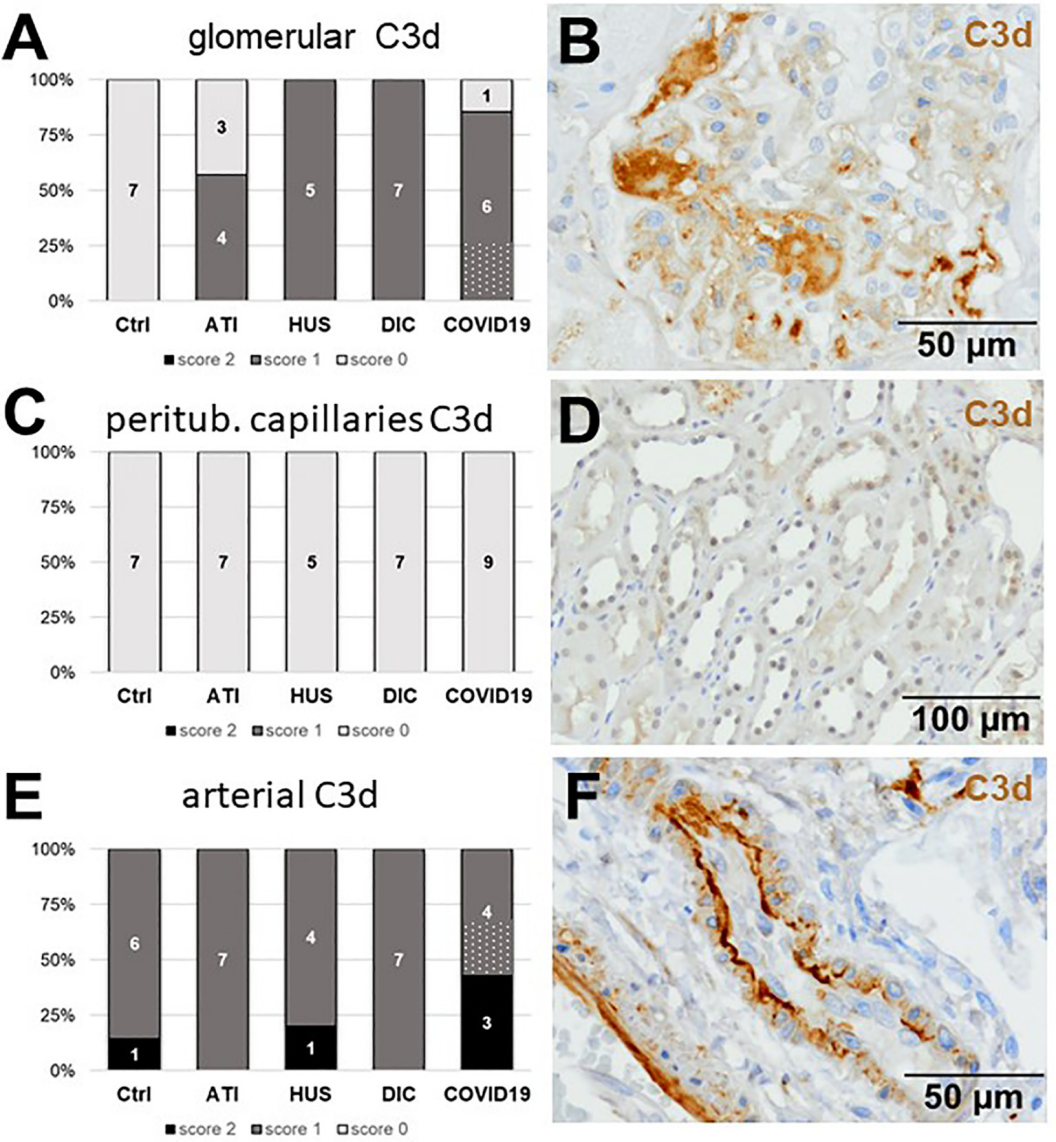
Frequency and vascular localization of C3d deposition in renal biopsies with COVID-19 compared to Ctrl, ATI, HUS, and DIC. Frequency and amount of C3d deposition were analyzed in glomerular capillaries **(A)**, peritubular capillaries **(C)** and renal arteries **(E)** in renal control biopsies taken 1 year after transplantation (Ctrl, n = 7), biopsies with acute tubular injury (ATI, n = 7), hemolytic uremic syndrome (HUS, n = 5), disseminated intravascular coagulation (DIC, n = 7) and COVID-19 (COVID-19, n = 9) using immunohistochemistry and semi-quantitative scoring. The proportion of C3d-positive stained cases that belongs to cases with comorbidities with known involvement of complement activation is marked by hatched bars representing the COVID-19 cohort. Representative pictures of COVID-19 biopsies positive for C3d were shown for glomerular (**B**, brown staining), peritubular (**D**, brown staining) and arterial localization (**F**, brown staining).

**Figure 9:**
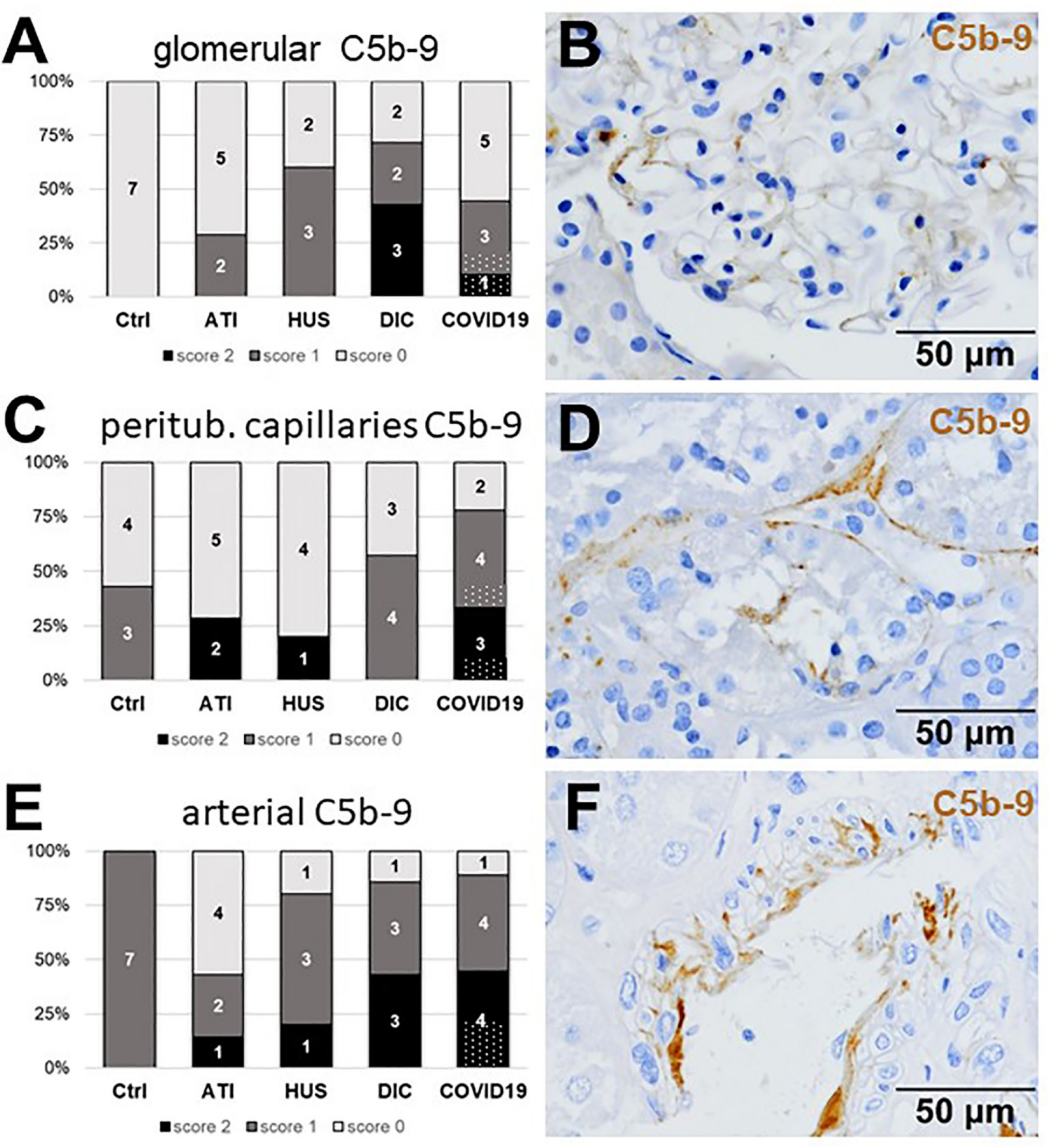
Frequency and vascular localization of C5b-9 deposition in renal biopsies with COVID-19 compared to Ctrl, ATI, HUS, and DIC. Frequency and amount of C5b-9 deposition were analyzed in glomerular capillaries **(A)**, peritubular capillaries **(C)** and renal arteries **(E)** in renal control biopsies taken 1 year after transplantation (Ctrl, n = 7), biopsies with acute tubular injury (ATI, n = 7), hemolytic uremic syndrome (HUS, n = 5), disseminated intravascular coagulation (DIC, n = 7) and COVID-19 (COVID-19, n = 9) using immunohistochemistry and semi-quantitative scoring. The proportion of C5b-9–positive stained cases that belongs to cases with comorbidities with known involvement of complement activation is marked by hatched bars representing the COVID-19 cohort. Representative pictures of COVID-19 biopsies positive for C5b-9 were shown for glomerular (**B**, brown staining), peritubular (**D**, brown staining) and arterial localization (**F**, brown staining).

As first complement factor, we detected the glomerular deposition of MASP-2, a serine protease, that complexes with the lectin pathway initiators (MBL, ficolins, and collectins) and becomes activated upon binding to the lectin ligand and found it in 2/9 of the COVID-19 biopsies. Compared to COVID-19 glomerular MASP-2 expression was higher in DIC, comparable in Ctrl, slightly lower in ATI and did not occur at all in HUS (**Figures 5A, B**). Additionally, in peritubular capillaries and renal arteries, MASP-2 was detected in only 22% 2/9 of cases from patients with COVID-19 respectively, but could otherwise only be detected in renal arteries of HUS and in peritubular capillaries in HUS and ATI groups and not at all in the Ctrl biopsies (**Figures 5C–F**). Next, we studied the renal deposition of C1q, which is part of the classical pathway (**Figure 6**). We rarely detected C1q in glomerular and peritubular capillaries in a single COVID-19 biopsy (1/9), which was comparable to Ctrl. In HUS and DIC renal biopsies it was present in the glomeruli of all biopsies and peritubular capillaries in 40% to 60% of the biopsies (**Figures 6A–D**). In COVID-19, C1q deposition in the renal arteries was seen in 4/9 of biopsies (**Figures 6E, F**) and even more commonly in HUS and DIC (**Figure 6E**). In Ctrl C1q deposition was slightly lower compared to COVID-19 while renal biopsies of patients with ATI showed no C1q deposition at all (**Figures 6A, C, E**). Next, we analyzed fragments of C3 that are formed by all complement pathways: C3c, a stable activation fragment and C3d an activation fragment that is able to covalently bind to surfaces. The stable C3 fragment C3c was detectable in the glomeruli of 4/9 of the COVID-19 biopsies, while glomerular C3c signals were comparable in ATI and more frequent and stronger in HUS and DIC and lower in Ctrl (**Figures 7A, B**). In peritubular capillaries, C3c was visible in 2/9 of COVID-19 biopsies, one of them at high intensity, and was thus comparable in frequency to ATI and HUS (**Figures 7C, D**). Only in DIC peritubular C3c was present in 6/7 of biopsies but also occurred in 4/7 Ctrl biopsies (**Figure 7C**). In renal arteries, the deposition of C3c was common in all groups and showed less intensity in Ctrl while other groups frequently showed intense staining (**Figures 7E, F**). C3d,. was detectable in the glomeruli of 8/9 of COVID-19 biopsies, while glomerular C3d signals were lower in ATI and completely lacking in Ctrl (**Figures 8A, B**). In contrast, more frequent glomerular C3d deposition was detected in HUS and DIC (**Figures 8A, B**). In peritubular capillaries, C3d was negative in all investigated groups (**Figures 8C, D**). In renal arteries, the deposition of C3d was common in all groups but strongest staining was observed in COVID-19 (**Figures 8E, F**). Interestingly, the complement split product C4d, that was commonly used to assess whether antibodies are participating in antibody-mediated rejection of renal transplants, was not detected in any of the kidney samples tested; neither in biopsies of COVID-19 patients nor in the controls (**Supplemental Figure 3**, **Table 2**). Finally, we studied C5b-9 deposition in renal specimens, the membrane attack complex formed in the terminal complement pathway (**Figure 9**). While C5b-9 was lacking in Ctrl and low in the glomeruli of ATI, it was deposited in four of nine COVID-19 cases, sometimes heavily (**Figures 9A, B**). The most intense deposition of C5b-9 was observed in DIC, while in HUS 3/5 of biopsies were also positive but showed only weak glomerular staining (**Figure 9A**). In contrast, peritubular C5b-9 was most common in COVID-19 (**Figures 9C, D**); in comparison Ctrl and DIC cases showed comparatively less C5b-9 and HUS showed less positive staining (**Figure 9C**). Comparable arterial C5b-9 deposits with light to intense staining were observed in COVID-19 and DIC, while in HUS arterial C5b-9 was similarly frequent but showed less intense staining (**Figure 9E**). Similar to most other complement components in this study, C5b-9 in renal arteries was lowest in ATI (**Figure 8E**) while Ctrl showed positive staining in all cases but with less intensity (**Figure 9E**). In summary, COVID-19 leads to endothelial cell damage in the kidney with the accumulation of platelets and significant activation of the complement system (summarized in **Table 2**), which might at least in part occur *via* the lectin pathway.

### Tubular Complement Activation Was Highest in Kidneys of Patients With COVID-19 and Restricted to C3c, C3d and C5b-9

**Figure 10:**
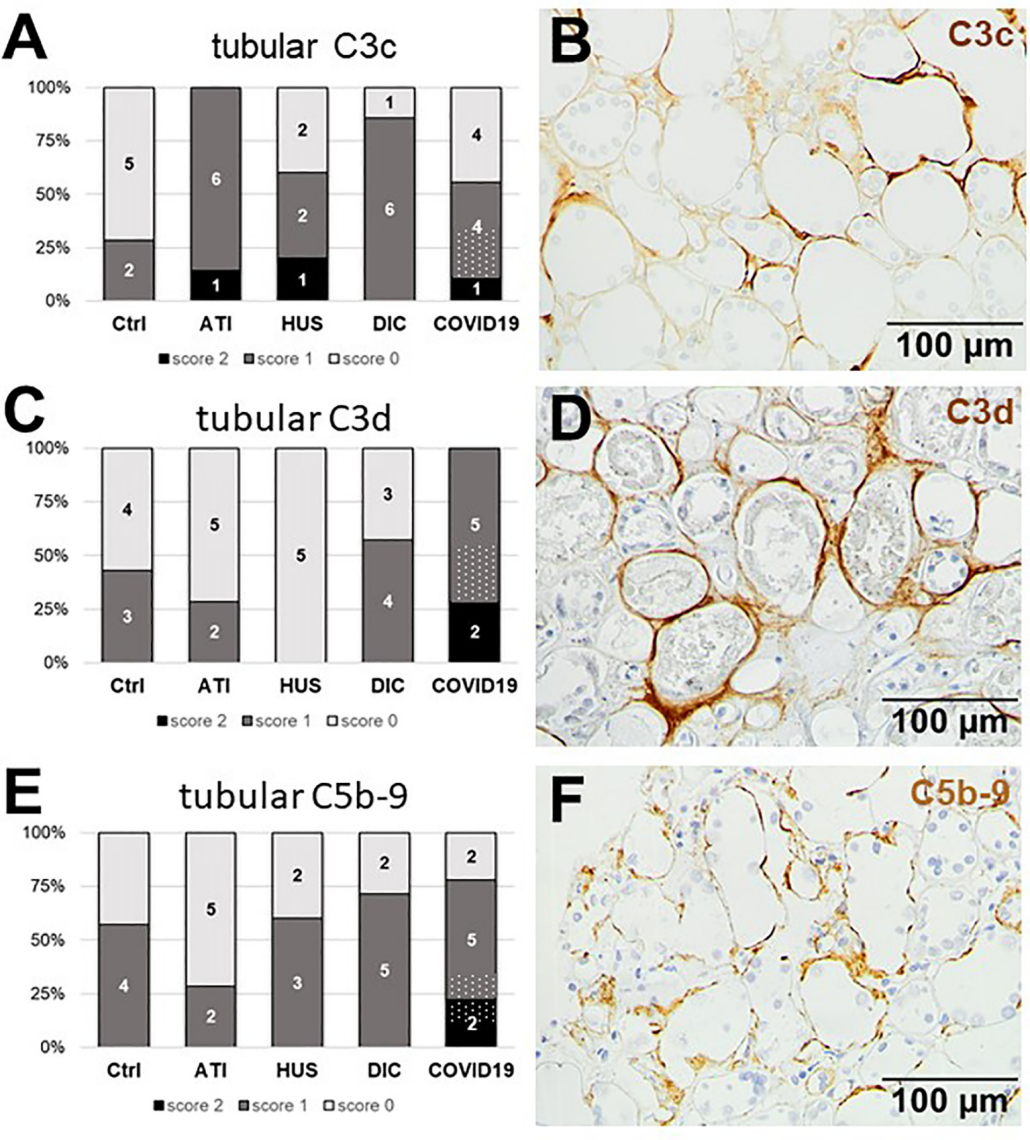
Frequency and tubular localization of C3c, C3d, and C5b-9 deposition in renal biopsies with COVID-19 compared to Ctrl, ATI, HUS and DIC. Frequency and amount of tubular C3c **(A)**, C3d **(C)**, and C5b-9 **(E)** deposition were analyzed in renal control biopsies taken 1 year after transplantation (Ctrl, n = 7), biopsies with acute tubular injury (ATI, n = 7), hemolytic uremic syndrome (HUS, n = 5), disseminated intravascular coagulation (DIC, n = 7), and COVID-19 (COVID-19, n = 9) using immunohistochemistry and semi-quantitative scoring. The proportion of complement-positive stained cases that belongs to cases with comorbidities with known involvement of complement activation is marked by hatched bars representing the COVID-19 cohort. Representative pictures of COVID-19 biopsies positive for tubular C3c (**B**, brown staining), C3d (**D**, brown staining), and C5b-9 (**F**, brown staining) were shown.

Since we observed tubular injury in all biopsies from patients with COVID-19, we next investigated complement deposition in the tubular compartment and detected complement deposition along the tubular basement membranes (**Figures 10B, D, F**). No tubular deposition could be detected for MASP-2 and C1q in our cohort (data not shown). Tubular C3c, was detected in 5/9 biopsies from COVID-19 patients, while tubular C3c deposition in HUS was comparable and more frequent in ATI and DIC (**Figure 10A**). In contrast, tubular C3d was detectable in all COVID-19 biopsies and showed the most frequent and intense staining compared to all other groups (**Figure 10C**). Interestingly, the C3d staining was strongest in kidneys from patients who died due to severe COVID-19 infection, indicating that complement mediated tubular damage is might be dependent on the severity of the COVID-19 disease. Tubular C5b-9 deposits could also be detected in 7/9 biopsies of patients with COVID-19 and again showed the highest intensity compared to the other groups (**Figure 10E**).

In summary, when the compartment-specific scores for all stainings were added up to a total score for the respective factor, biopsies of COVID-19 patients contained the highest number of CD61-positive platelets compared to the other groups (**Table 2**). This parallels with the deposition of C3d, C5b-9 and on a lower level with the deposition of MASP-2 (**Table 2**). C1q deposition in biopsies from patients with COVID-19 was comparable with Ctrl and even higher in HUS and DIC (**Table 2**), indicating that in COVID-19 renal complement was not activated *via* the classical but by the lectin or alternative pathway.

## Discussion

Complement factors were major components that clogged CRP adsorber columns during the treatment of critically ill patients with COVID-19 prompting further studies on a role of complement in COVID-19 infection. C5b-9 and the complement cleavage product and anaphylatoxin C5a were increased in the plasma of patients with moderate and severe disease compared to healthy controls (20). Furthermore, lung epithelial cells infected with SARS-CoV-2 highly expressed the complement factors C1r, C1s, C3 and factor B (22). Studies by immunohistology on complement-mediated microvascular damage showed deposits of MASP-2, C4d, and C5b-9 in the lungs and skins of severely ill patients (21). In a preprint study Gao et al. demonstrated, that the SARS-CoV-2 nucleocapsid protein bound to MASP-2 and activated complement; blockade of this interaction improved the survival of mice with COVID-19 nucleocapsid potentiated LPS-induced pneumonia (23). In our study in kidney biopsies from patients with COVID-19, we for the first time detected enhanced renal complement deposition in vascular beds and tubuli supporting the idea that the complement system is a potential mediator of COVID-19–induced kidney injury. The observed kidney damage in COVID-19 biopsies was primarily acute kidney injury (AKI) with mild to severe tubular injury and in rare cases glomerular thrombi. This is consistent with the observations of other histopathological studies examining kidneys of the patients with COVID-19 and identifying AKI in 37% to 98% of all examined cases (9–11). Interestingly, especially the tubular deposition of C3d was highest in biopsies from patients with COVID-19 and seem to be dependent on the severity of the disease, supporting the role of complement as a mediator of tubular damage. In addition, we investigated endothelial cell loss in COVID-19 biopsies. The endothelium plays an important role in the pathogenesis of COVID-19–mediated tissue damage, both as an effector contributing to inflammation and thrombosis, and as a target organ, whose dysfunction may contribute to poor outcome (24). In an autopsy study from patients who died of COVID-19, pulmonary vessels showed widespread thrombosis, capillary microthrombi and increased neovascularization (25). In renal biopsies we observed a reduction of the endothelial cell marker ERG, similar to the endothelial damage in ATI, but less pronounced than in DIC. Renal capillarization was highest in HUS, indicating that the endothelial cell damage already started to be repaired by high proliferative activity. In contrast to reports in the lung (25), microthrombi and endothelial proliferation were rare in COVID-19 renal biopsies. However, CD61-positive platelets in peritubular capillaries were highest in COVID-19 biopsies compared to all other groups. In comparison with other renal diseases with distinct endothelial cell injury, like HUS or DIC, we demonstrate similar complement activation in COVID-19 renal biopsies with some differences in the involved pathways. The lectin pathway can be activated by sugar residues bound to pattern recognition molecules followed by activation of MASP-2 complexes with collectin 11. In ischemic kidneys, collectin 11 recognized an abnormal L-fucose pattern on tubular cells and consequently activated the lectin pathway (26). Interestingly, in tubuli from COVID-19 biopsies we observed a higher collectin 11 mRNA expression compared to 1 year biopsies, HUS and ATI controls (**Supplemental Figure 1**) and MASP-2 deposition in some of the COVID-19 cases, indicating that activation of the lectin pathway in COVID-19 was not restricted to lung or skin (21). Activation of the classical pathway can be mediated for example by natural IgM antibodies that recognize viral antigens or neo-antigens exposed on damaged host tissues (27). C1q, the starter of the classical pathway, was more frequently detected in COVID-19 as compared to ATI but, nevertheless, rarely detected in glomeruli and peritubular capillaries compared to HUS and DIC cases. However, the highest C1q deposition in COVID-19 biopsies was seen in renal arteries. C3 cleavage products occur in all three complement activation pathways and were detected as the stable cleavage product C3c and C3d, which can bind covalently to cell surfaces. Interestingly, deposition of these C3 cleavage products were not similarly deposited in the renal biopsies. C3d was highest in biopsies from patients with COVID-19, C3c was also increased compared to controls but even higher in HUS and DIC. Differences in staining patterns are may be due to the specificity of the used antibodies. While the anti-C3c antibody also recognize C3 the anti-C3d do not detect intact C3. C5b-9, as a marker of the terminal complement cascade, was strongly and similarly detectable in glomerular capillaries and even more strongly deposited in the peritubular capillaries than in HUS and DIC biopsies. Significant C5b-9 deposition was also described in lung and skin biopsies from patients with COVID-19 (21).

In summary, in our study complement activation in COVID-19 was not restricted to a specific activation pathway. Complement was reportedly activated by all three known pathways during COVID-19 infection (28). It is conceivable that complement activation can occur both through direct interaction with the SARS-CoV-2 virus or indirectly by tissue damage and dying cells. Presumably, the complement activation in kidneys of COVID-19 patients observed in our study was rather indirect, since we could hardly detect viruses by in-situ hybridization and even fewer pathogens by immunohistochemistry so that we cannot exclude that these are unspecific background signals. This goes inline with findings of other groups, which have also not found robust signals of SARS-CoV-2 in the kidneys (9–11). In contrast, in other studies SARS-CoV-2 was detected in the kidney (5, 7, 8), so that we cannot exclude the possibility that SARS-CoV-2 was present in the kidney at an earlier time interval before biopsy was taken and that complement was activated directly by the virus. Our own and data of other groups showed that complement activation might be an important pathomechanism of tissue damage in COVID-19 opening the possibility of treatment with complement inhibitors. Anecdotal reports of the successful treatment of seriously ill patients with COVID-19 employing the C5 inhibitor Eculizumab (29) or the C3 inhibitor AMY-101 (30) have already been published. Clinical studies with complement inhibitors in patients with COVID-19 will show the efficiency of this treatment. Several clinical trials are currently ongoing to investigate the protective effects of purified/recombinant complement regulators (e.g. C1-esterase inhibitor) or complement inhibitors directed against MASP-2, C3, C5, or the C5a receptor on COVID-19 outcomes ranging from changes in oxygenation to mortality (31)(https://www.trialsitenews.com/category/masp-2/).

## Limitation of the Study

Our study is limited by the small number of COVID-19 renal biopsies and the different co-morbidities of the investigated patients with kidney disease. At least 3 of the comorbidities are also known for the occurrence of complement activation, namely ANCA-associated GN, FSGS and infectious GN. To distinguish between COVID-19–related complement activation and complement activation that might be related to the diagnosed renal disease, the proportion of positively stained cases that belongs to the 3 above mentioned cases is marked in the figures by hatching. Indeed, we frequently but not necessarily always detected complement deposition outline biopsies with these diseases. However, the percentage of complement-positive cases in COVID-19 with known potential complement involvement represented on average 30.2% of all positive COVID-19 cases. Although we cannot rule out that the observed complement deposition in the cases with the above mentioned kidney diseases was due to this comorbidity and not to the COVID-19 infection, the renal complement deposition was by far not limited to these cases. Renal thrombi were only found in one investigated post-mortem biopsy, therefore it remains unclear if thrombi only occur in severely diseased kidneys or during peri-mortal processes. Since the antibody used for detection of MASP-2 also recognize the MASP-2 splice variant MAp19, lacking the catalytic domain, we cannot distinguish between complement activating MASP-2 and Map19. Although our initial observation that complement factors are an essential component of plasma clots in CRP adsorbers in critically ill patients with COVID-19 led to the studies in the kidney, the role of complement in the clotting process remains open and needs further investigation.

In conclusion, we observed marked complement deposition in kidneys of patients with COVID-19 similar to other known renal diseases with a distinct endothelial injury. Complement deposition could be also detected on tubular basement membranes in biopsies from patients with COVID-19. Therefore, we speculate that complement is, most likely involved in vascular and tubular kidney damage in COVID-19. Specific complement inhibition might thus be a promising treatment option to prevent deregulated complement activation and subsequent collateral tissue injury. Further studies with larger biopsy numbers are necessary to elucidate the involvement of the complement system in COVID-19–induced renal damage.

## Data Availability Statement

The original contributions presented in the study are included in the article/**Supplementary Material**. Further inquiries can be directed to the corresponding author.

## Ethics Statement

The studies involving human participants were reviewed and approved by Ethics Committee of Friedrich-Alexander-University Erlangen-Nürnberg (Reference No. 4415). Written informed consent for participation was not required for this study in accordance with the national legislation and the institutional requirements.

## Author Contributions

EV and FP conducted experiments, analyzed data, and wrote the manuscript. TR carried out immunohistological staining. H-MJ and KÜ generated the anti-SARS-CoV-2 spike antibody, supplied transfected cells, and proofread the paper. GL performed MALDI-TOF. AS isolated proteins from plasma adsorber material. MH conceived and designed the plasma adsorber analysis and revised the manuscript. KA, MB-H, and CD conceived and designed the study, analyzed data, and wrote the manuscript. All authors contributed to the article and approved the submitted version.

## Funding

This study was funded by the Deutsche Forschungsgemeinschaft (DFG, German Research Foundation), project number 387509280, SFB 1350 and supported by the DEFEAT Pandemics autopsy platform of the Federal Ministry of Education and Research (BMBF).

## Conflict of Interest

Author AS was employed by the company Pentracor GmbH.

The remaining authors declare that the research was conducted in the absence of any commercial or financial relationships that could be construed as a potential conflict of interest.
