# Supplementary figures and images for: Complement Activation in Kidneys of Patients With COVID-19
Source: Front Immunol. 2021 Jan 29;11:594849. doi: 10.3389/fimmu.2020.594849 (PMC7878379; doi:10.3389/fimmu.2020.594849)

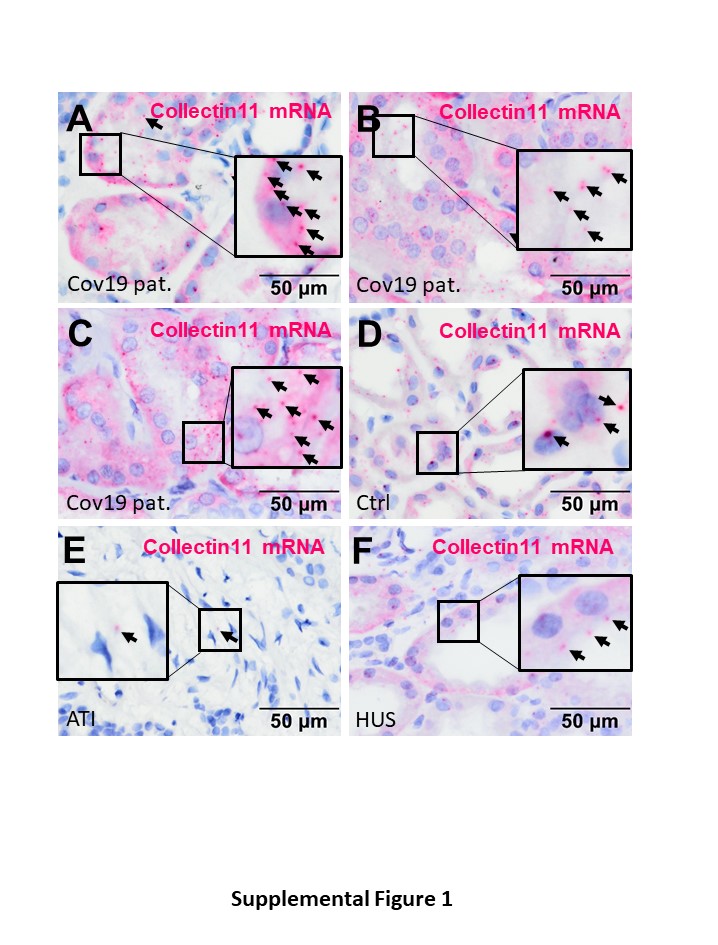

Supplement: Supplementary file 1 [file Image_1.jpeg]

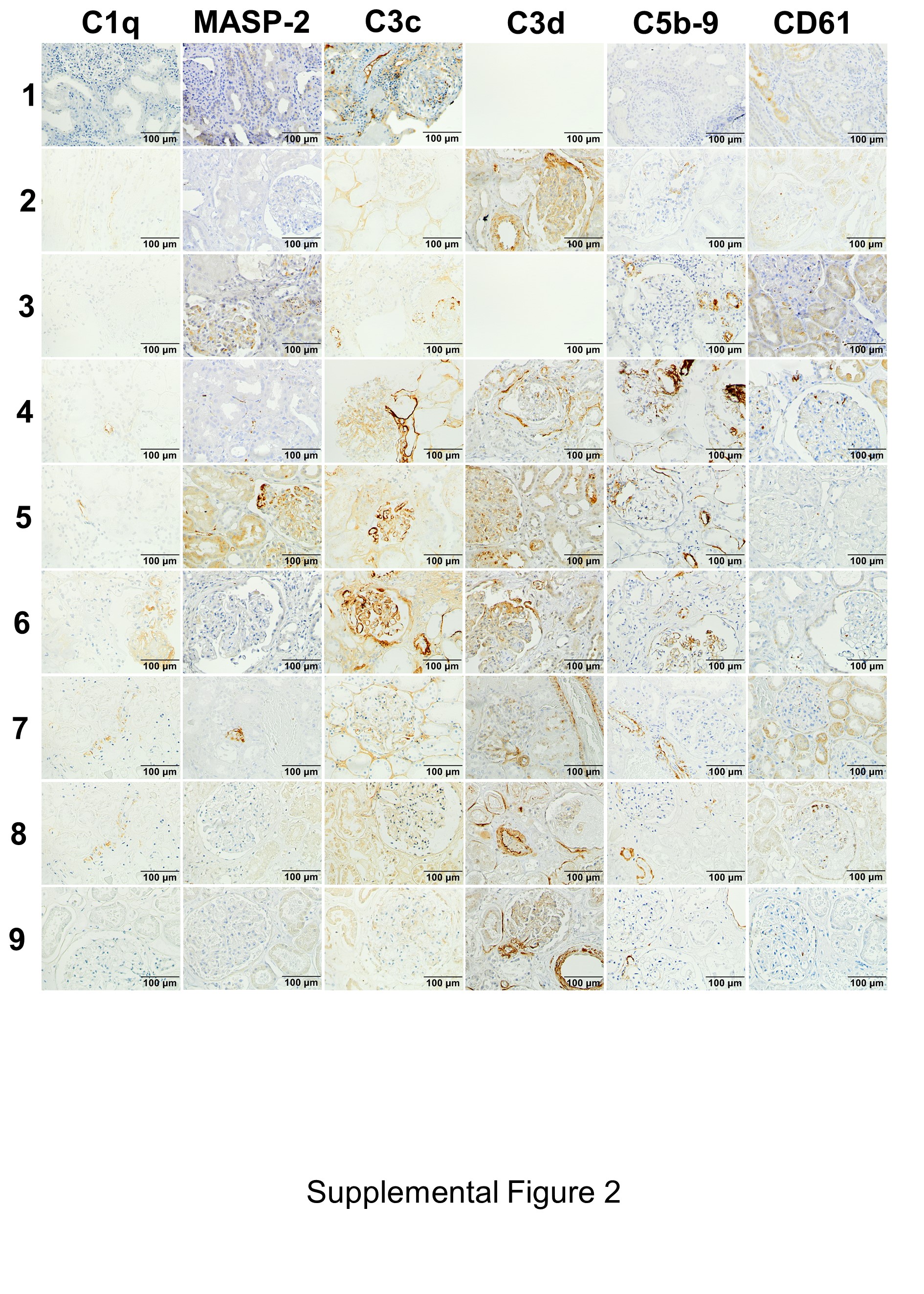

Supplement: Supplementary file 2 [file Image_2.jpeg]

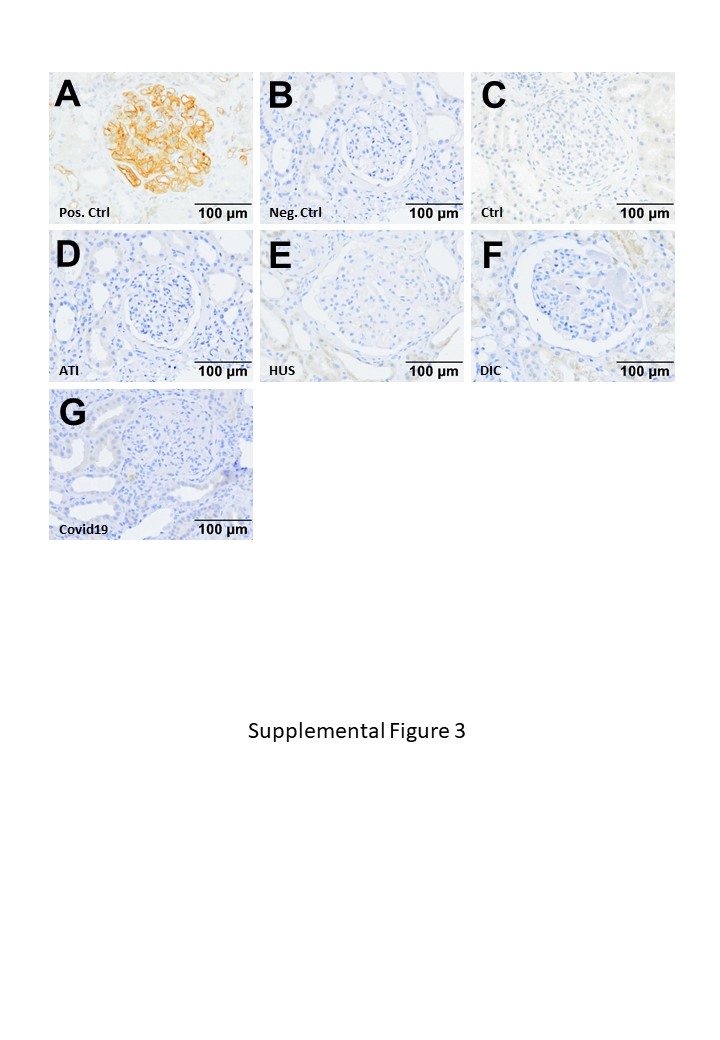

Supplement: Supplementary file 3 [file Image_3.jpeg]
